# Prophesies and Disclosures in Dreams

**Published:** 1886-12

**Authors:** 


### HALL'S JOURNAL OF HEALTH

TRUTH DEMANDS NO SACRIFICE; ERROR CAN MAKE NONE.

VOL 33. DECEMBER, 1886. No. 12.

SPECIAL.—*Hereafter this* JOURNAL *will be issued punctually on the first of every month, instead of the fifteenth, as heretofore.*

### PROPHESIES AND DISCLOSURES IN DREAMS.

All who have given the subject attention must agree with Mr. Owen, that, if the accounts of some of the most remarkable dreams are to be relied upon, there are phenomena and laws connected with dreaming which have never yet been explained, nor scarcely investigated. In the last two issues of the JOURNAL we have called the attention of our readers to this interesting subject, with a view of illustrating the position which seems to us the only tenable one, that we are frequently influenced in our sleep by intelligencies, outside of ourselves, capable of imparting to us knowledge of which we had no previous conception, and putting us in the way of making inventions and discoveries which our unaided minds would have never thought out. Jacquard affords us an instance of this, whose simple yet intricate invention of embelishing fabrics with figures, forms and curious devices, in the process of weaving, is one of the most curious as well as useful known to the arts. Hundreds of others have bequeathed to the world similar legacies which have aided its advancement and added to its wealth. Not only this, but hidden treasure has been revealed, ruinous plots exposed, and lives saved by timely dream-warnings sometimes, more than once repeated to ensure their proper heeding.

We have an acquaintance who has made several inventions of a purely scientific description, which have been widely introduced, yet he informs us that in each case all the details of the invention, as well as the mechanical contrivances which make it available in practice, were given to him so clearly as to enable him to reproduce them in drawings, without the aid of sketch or model.

Tradition and history abound in narratives of dreams, not only of inventions and discovery, but also of warning and prophecy, which were afterwards confirmed or followed to profitable account. We shall find space to narrate only a few of the most remarkable ones, but sufficient with what have been given in illustration of our text, to satisfy the reader of the verity of these, the commonest of phenomena, which no pen has as yet been able fully to illustrate. Nearly all the best known Greek and Roman philosophers treated of this subject in its various aspects, and some of them ascribed to dreams a divine or spiritual origin.

Cicero, in his " De Divinatione," holds that in the visions of the night men occasionally receive more than is taught them throughout all the waking hours of day. This author, as well as other contemporary writers, gives us an ascount of the dream of Simonides, a Greek poet of a period more than 500 years B. C., who, while on his way to a distant country, discovered the dead body of a stranger, and learning that no friends were at hand to claim the corpse, had it decently interred at his own expense, and then prepared to continue his journey. But the night before his contemplated departure, the unknown, whose body he had favored with burial, appeared to him in his sleep and warned him not to embark on the vessel he had chosen, else certain destruction should overtake him. Simonides was so impressed by the appearance that he heeded the injunction, and a few weeks later learned that the ship upon which he had arranged to take passage had gone to the bottom of the Mediterranean with all on board.

The unfortunate Condorcet, the famous mathematician, although a materialist, relates of himself that when engaged in some profound and puzzling calculations he was often forced to leave them uncompleted and retire to rest, and that often the concluding steps were indelibly impressed on his mind in a dream. Dr. Franklin also states that intricate political questions were frequently made clear to him while wrapped in slumber. Ben Johnson, the well known dramatist of the seventeenth century, confessed to having been warned by a dream of the death of a favorite child.

The dream of the Prince of Conde is one that engages attention at once from the number of coincidences demanded to complete its verification. It was during the French religious war, in which the prince was the principal Protestant chief, and just before the battle of Dreux, that he beheld the vision in question. He dreamed that he had engaged in three successive battles, and had gained as many victories, costing the lives of his three leading enemies of the opposition—the Marshal of St. Andre, the Duke of Guise and the Constable of France. He himself mortally wounded, expired amid their corpses. The historical fact is that St. Andre perished at Dreux, the Duke of Guise at Orleans and the constable at St. Denis, while the Prince of Conde himself met his death after them at the battle of Bassac.

The following will particularly interest our lady readers, as indicative of a superior direction against the acquisition of an establishment at the sacrifice of the affections.

In a small town of Central France, a few years ago, Angele Bobin, a young girl of humble parentage, but remarkable for her beauty and grace, was urged by her parents to accept an advantageous offer of marriage which was distasteful to her. The matter being warmly pressed, she earnestly prayed for spiritual guidance. This was followed by a dream, in which a young man passed before her in a traveler's dress, wearing spectacles and a large straw hat. Regarding the vision as an answer to her prayer, and being encouraged by her teacher, to whom she related it, she firmly resisted the projects of her parents, who were induced to abandon them. Some time afterward, at a village gathering, she recognized the young traveler of her dream in the person of M. Emile de la Bedolliere, one of the editors of the *Siecle*, a Parisian journal. They fell in love at first sight, as the saying is, and were soon married. This was in 1833. In a letter to Dr. Marcario, dated Paris, 13th December, 1854, the husband certified to the accuracy of the foregoing statements, with some additional particulars confirmatory of them.

The dream of Monsieur Bach, a Parisian of distinguished family, which we have condensed from the account of the late Robert Dale Owen, is one of the most remarkable, as having a historical interest, of which we have any account. A son of this gentleman had purchased at a bric-a-brac shop in Paris, in 1865, an old and curiously ornamented *spinet*; an instrument with wire strings and keys, similar in many respects to our modern piano. Upon a critical examination of his son's purchase, M. Bach discovered, much to his delight, that it was made in Rome in the year 1564, its antiquity adding greatly to its value, in the estimation of curio-mongers. On retiring to rest M. Bach dreamed that a handsome young stranger appeared to him, elegantly attired in the ancient costume of the French court. Doffing his high pointed crowned white plumed hat, the stranger advanced, with great courtesy toward the sleeper and thus addressed him :

"The Spinet you have belonged to me. I often played upon it to amuse my master, King Henry. In his youth he composed an air with words which he was fond of singing while I accompanied him. Both words and air were written in memory of a lady whom he greatly loved. He was separated from her, which caused him much grief. She died, and in his sad moments he used to hum this air. I will play it to you and I shall take means to recall it to your recollection, for I know you have a poor memory." Thereupon the visitor sat down to the Spinet accompanying himself as he sang the words. They were so touching that M. Bach awoke in tears. It was then two o'clock at night. Again composing himself to sleep, he remembered nothing more until daylight, when, to his astonishment, he found lying on his bed, the song of his dream written in the musical characters and orthography in vogue three hundred years before, and purporting to have been composed by Henry III. of France. The sheet upon which it was written was one of M. Bach's, on the reverse side of which was one of his own compositions written by him only the previous day, and put away in his escritoire.

It was some weeks after the events above narrated, that M. Bach, sitting by himself, felt a nervous trembling of his arm, and upon taking a pencil in his hand, soon lost consciousness, and wrote, "King Henry, my master, who gave me the spinet you now possess, had written a four line stanza on a piece of parchment which he caused to be nailed on the case, when one morning he sent me the instrument. Some years afterward, having to travel and take the spinet with me, fearing that the parchment might be torn off and lost, I took it off, and for safe-keeping put it in a small niche, on the left of the key-board, where it still is."

This communication was signed Baldazzarini, and following it was the stanzas alluded to. After a long and apparently hopeless search, which occupied more than an hour, the instrument was further taken to pieces, and underneath the key-board and some of the hammers, a bit of parchment was found, 11½x2¾ inches in size, upon which was written in a bold, dashing hand, occupying four lines the presentation stanzas, signed Henry, which, upon being critically examined by competent archiologists, was pronounced to be in the handwriting and signature of Henry III., a literal translation of which is given by Mr. Owen as follows :

> I, the King Henry III, present this Spinet,
> To Baltasarini, my gay musician ;
> But if he finds it poor-toned, or else very simple,
> Still, for my sake, in its case let him preserve it.

It was only after a long search in different libraries that Mr. Owen was able to find any account of Baltazarini, but a French Dictionary of Musicians in the Athenaum Library of Boston, finally furnished it, by which it appears that he was brought from Piedmont to the Court of Queen Catherine de Medicis in 1577, and was entrusted by Henry III. with the management of court fetes, and that in this capacity he was the first to devise dramatic entertainments combined with singing and dancing, identical with our modern opera.

We have been constrained to omit many of the details of this authentic narrative, which may be said to furnish *record* evidence of the communion of spirit and mortal in dreams, as also the subjection of a human mechanism to the will of the communicating spirit in other ways.

The story of Henry's love for the beautiful Princess Marie de Cleves, whom the differences in religion prevented him from marrying, is in itself a romance from whose disappointment the king was never able to overcome, hence the pathetic love ditty which he was wont to sing in his hours of melancholy retrospection, to the accompaniment of his favorite musician. The story is told at length by Mr. Owen in his "Debatable Land," with the words of the song complete and fac-simile illustrations of the mysteriously written music, and also of the parchment found in the old spinet, which had so securely kept the secret for nearly three centuries.

In late numbers of the JOURNAL we have given the particulars of several instances of the recovery of lost treasure, through information derived in dreams, after all previous searches for it had failed, and many are the accounts of the preservation of life from the assassin's hand, and the tracing of crime through the same means.

We will close this article, which at best is only a rapid glance of a subject worthy of a more thorough treatment, with the account of a dream nearer home as well as of recent date, with the circumstances of which we have been made familiar. A lady of our acquaintance, residing in Brooklyn—we will call her Mrs. Smith—several years ago, was persuaded to receive into her family, and care for a male infant, the mother of which was quite well to do, but for some unexplained reason, preferred such an arrangement. For a considerable time, everything was satisfactory to both parties. The visits of the mother were frequent, and payments punctual. This, however, did not continue beyond a few months, and at length the mother was lost sight of altogether. Still Mrs. Smith, who in the meantime had formed a mother's fondness for the little one, continued to care for him. At length she became a widow, and this, with other misfortunes, made it quite necessary that she should receive the sum due to her. Where to find the mother was now the question.

Years had passed since their last meeting, during which she had tended and nursed the child in sickness and in health, and he had a full measure of diseases incident to early childhood, including an unusually severe attack of scarlet fever, the effects of which will never leave him. In a state of great anxiety Mrs. S. dreamed one night that by calling at a certain house, the location of which was minutely described, she would meet with a man who would direct her to a large house nearby, where she would be able to learn the address and whereabouts of the recusant mother, who had in the meantime married, giving her present name. This dream made so strong an impression upon Mrs. S's. mind that she determined to follow out its directions. She accordingly repaired to the intersection of the two streets indicated, which, although strange to her, were readily recognized as the ones of her dream ; thence following the course indicated, she counted the houses till she reached the right one, and rang the door bell. Here she was met by a man, whose uniform showed him to be a letter-carrier, and on making the proper enquiries, she learned that letters for the person of whom she was in quest were delivered at a large house which the carrier pointed out, and upon enquiring there Mrs. Smith was directed to her then residence, and the two were brought face to face in personal interview, which resulted, after some negotiation of rather an embarrassing nature, to the pecuniary advantage of the dreamer.

It is with some reluctance that we feel compelled to relinquish a subject for the time being which offers so many inducements, but we reserve the right to return to it occasionally as new evidences of an interesting character are presented to us, and such we invite, for our future labors will be no less in the field of matter than that of spirit, in so far as they affect our physical and moral health, happiness and well-being.

